# Supplementary material for: Electric Fields Elicit Ballooning in Spiders
Source: Curr Biol. 2018 Jul 23;28(14):2324–2330.e2. doi: 10.1016/j.cub.2018.05.057 (PMC6065530; doi:10.1016/j.cub.2018.05.057)
Supplement: Document S1. Figure S1 and Table S1 [file mmc1.pdf]

Current Biology, Volume *28*

Supplemental Information

Electric Fields Elicit Ballooning in Spiders

Erica L. Morley and Daniel Robert

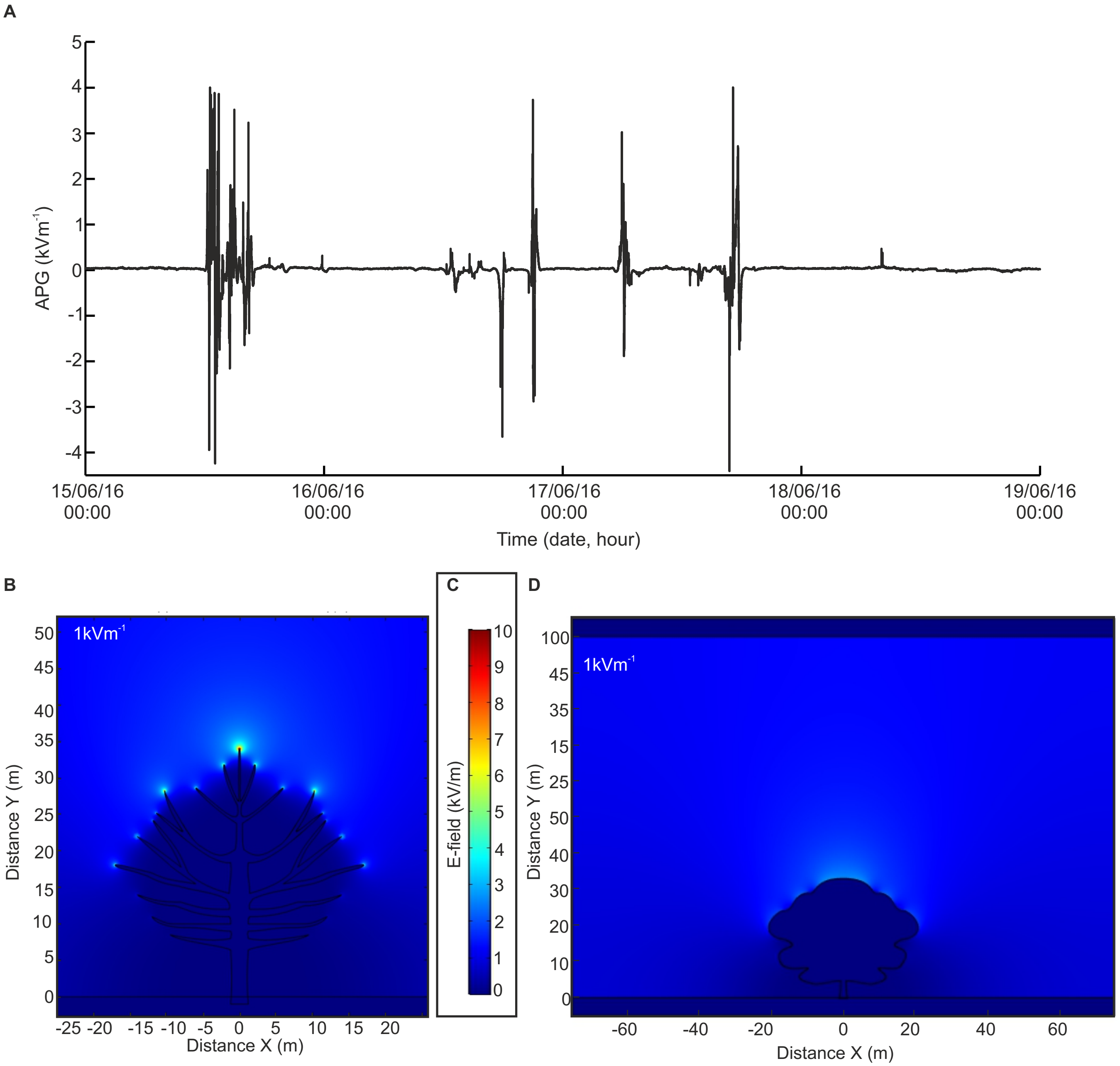

Figure S1. Further electric field quantifications, related to Figure 1

A) Atmospheric potential gradient (APG) measured across 4 days using a field mill (Chillworth JCI131) at the University of Bristol School of Veterinary Sciences, Langford. B) FEA model detailing the e-field around geometrically sharp tree branches in an APG strength of 1kVm^{-1}.C) Scale bar for (B) and (D). D) FEA model of e-field enhancement around a geometrically domed oak tree in an APG strength of 1kVm^{-1}. The scale in figure S1 B-D are the same as the scale in figure 1B-D, for comparison.

**Distribution**	**Fixed factor**	**Level**	**Coefficient estimate**	**Standard error**	**Z value**	**p value**
Tiptoes						
Poisson	Field strength (reference $0Vm^{-1}$)	$1.25Vm^{-1}$	1.25	0.8	1.565	0.118
		$6.25Vm^{-1}$	2.91	0.72	4.025	<0.0001***
Dragline drops						
Poisson	Field strength (reference $0Vm^{-1}$)	$1.25Vm^{-1}$	1.04	0.35	2.952	0.003**
		$6.25Vm^{-1}$	1.61	0.33	4.873	<0.0001***

Table S1. GLMM statistical analysis, related to Figure 2

Results of GLMM fits to ballooning behaviour data. All models had spider identification number as a random factor with varied intercept and the same slope (1|spider identification) to prevent pseudoreplication of repeated measurements on the same animal.
