# Supplementary material for: Electric Fields Elicit Ballooning in Spiders
Source: Curr Biol. 2018 Jul 23;28(14):2324–2330.e2. doi: 10.1016/j.cub.2018.05.057 (PMC6065530; doi:10.1016/j.cub.2018.05.057)
Supplement: Document S2. Article plus Supplemental Information [file mmc3.pdf]

Current Biology

Report

# Electric Fields Elicit Ballooning in Spiders

## Highlights

- Spiders detect electric fields at levels found under natural atmospheric conditions
- Ballooning behavior is triggered by such electric fields
- Trichobothria mechanically respond to such electric fields, as well as to air flow
- Electric field and air flow stimuli elicit distinct displacements of trichobothria

## Authors

Erica L. Morley, Daniel Robert

## Correspondence

erica.morley@bristol.ac.uk

## In Brief

Morley and Robert show that spiders can detect electric fields and respond to this stimulus by attempting to balloon. They conclude that atmospheric electrostatics could provide forces sufficient for dispersal by ballooning in spiders and that hair-shaped sensors are putative electroreceptors.

Morley & Robert, 2018, Current Biology *28*, 2324–2330
July 23, 2018 © 2018 The Authors. Published by Elsevier Ltd.
https://doi.org/10.1016/j.cub.2018.05.057

CellPress

Current Biology
Report

# Electric Fields Elicit Ballooning in Spiders

Erica L. Morley[1,2,*] and Daniel Robert[1]
[1]School of Biological Sciences, Life Sciences Building, University of Bristol, 24 Tyndall Avenue, Bristol BS8 1TQ, UK
[2]Lead Contact
*Correspondence: erica.morley@bristol.ac.uk
https://doi.org/10.1016/j.cub.2018.05.057

## SUMMARY

**When one thinks of airborne organisms, spiders do not usually come to mind. However, these wingless arthropods have been found 4 km up in the sky [1], dispersing hundreds of kilometers [2]. To disperse, spiders "balloon," whereby they climb to the top of a prominence, let out silk, and float away. The prevailing view is that drag forces from light wind allow spiders to become airborne [3], yet ballooning mechanisms are not fully explained by current aerodynamic models [4, 5]. The global atmospheric electric circuit and the resulting atmospheric potential gradient (APG) [6] provide an additional force that has been proposed to explain ballooning [7]. Here, we test the hypothesis that electric fields (e-fields) commensurate with the APG can be detected by spiders and are sufficient to stimulate ballooning. We find that the presence of a vertical e-field elicits ballooning behavior and takeoff in spiders. We also investigate the mechanical response of putative sensory receivers in response to both e-field and air-flow stimuli, showing that spider mechanosensory hairs are mechanically activated by weak e-fields. Altogether, the evidence gathered reveals an electric driving force that is sufficient for ballooning. These results also suggest that the APG, as additional meteorological information, can reveal the auspicious time to engage in ballooning. We propose that atmospheric electricity adds key information to our understanding and predictive capability of the ecologically important mass migration patterns of arthropod fauna [8].**

## RESULTS AND DISCUSSION

In the early 1800s, two competing hypotheses were proposed to explain how ballooning animals become airborne, invoking (1) the aerodynamic drag from wind acting on the silk or (2) atmospheric electrostatic forces [9]. Aware of the prevailing arguments, Charles Darwin mused over how thermals might provide the forces required for ballooning as he watched hundreds of spiders alight on the Beagle on a calm day out at sea [10]. Darwin's observation, however, did not provide further evidence in support of either hypothesis. The physical force required for ballooning has since been attributed to aerodynamic drag at low wind speeds (<3 $ms^{-1}$) [4, 5, 11], yet the involvement of electrostatic forces in ballooning has never been tested. Several issues have emerged when models using aerodynamic drag alone are employed to explain ballooning dispersal. For example, many spiders balloon using multiple strands of silk that splay out in a fan-like shape. Instead of tangling and meandering in light air currents, each silk strand is kept separate, pointing to the action of a repelling electrostatic force [12]. Questions also arise as to how spiders are able to rapidly emit ballooning silk into the air with the low wind speeds observed in ballooning; the mechanics of silk production requires sufficient external forces to pull silk from spinnerets during spinning [13]. And, how do low wind speeds provide the high initial accelerations seen in ballooning takeoff [10]? Attempts to find weather patterns that predict the prevalence of ballooning have been made, but results remain inconsistent [14]. Mass ballooning events occur sporadically, and weather conditions on days with abundant aeronauts cannot be readily distinguished from days void of them. Although reports claim thermal air currents and temperature gradients on fair-weather days are the driving force [15–18], ballooning can be observed when skies are overcast, as well as in rainy conditions ([14, 15] and E.L.M, unpublished data). Humidity is potentially an important predictor [19, 20], but causal and testable explanations are lacking. One consistent predictor of ballooning is wind speed; spiders only take flight when wind speed is below 3 $ms^{-1}$ [11, 15, 17, 19–21], a very light breeze, but models show that these conditions should not allow large spiders to balloon, despite observation to the contrary [12].

In the early 20[th] century, atmospheric electricity was intensively studied, establishing the ubiquity of the atmospheric potential gradient (APG) [6]; from fair to stormy weather, an APG is always present, varying in strength and polarity with local meteorological conditions. Over a flat field on a day with clear skies, the APG is approximately 120 $Vm^{-1}$ (Figure S1). In more unsettled meteorological conditions, charged clouds passing overhead modify the APG, with rainclouds, storm clouds, and mist or fog generating APGs of several kilovolts per meter [6, 22, 23] (Figure 1A). Any electrically grounded, geometrically sharp structure protruding from this flat field will cause a substantial enhancement of local electric fields (e-fields) [24] (Figures 1C and 1D). Fundamentally, this is why lightning rods work to channel a safe, predictable, path for lightning to reach ground. Because they are rooted in the earth and contain a high proportion of water and electrolytes, plants tend to equalize to ground potential [25, 26], and the electric field strength surrounding leaves and branches, due to their sharp geometry, can reach many kilovolts per meter [25–27] (Figures 1B–1E). For example, in mildly unsettled weather (APG of 1 $kVm^{-1}$), the

 © 2018 The Authors. Published by Elsevier Ltd.
This is an open access article under the CC BY license (http://creativecommons.org/licenses/by/4.0/).

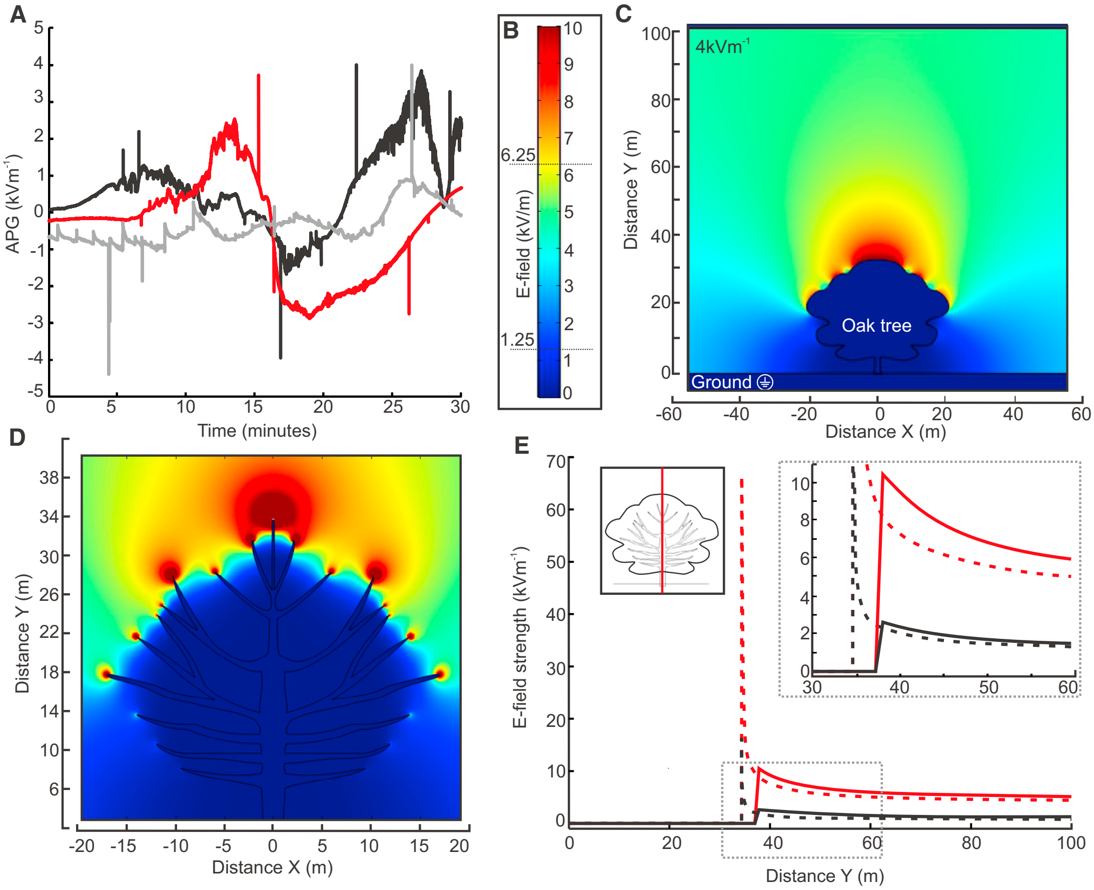


**Figure 1. Quantifying Electric Fields in Nature**

(A) Atmospheric potential gradient (APG) measured for 30 min periods across 3 days using a field mill (Chillworth JCI131) at the University of Bristol School of Veterinary Sciences, Langford. Colors depict recordings from different days in June 2016.

(B) Scale bar for (C) and (D).

(C) Finite element analysis (FEA) model of electric field (e-field) enhancement around a geometrically domed oak tree in an APG strength of 4 kVm$^{-1}$.

(D) FEA model detailing the e-field around geometrically sharp tree branches in an APG strength of 4 kVm$^{-1}$.

(E) Two-dimensional plot of the e-field along cut lines (red; left inset) of (C) oak modeled as geometrically domed (solid) and (D) branches (dashed) in an APG of 4 kVm$^{-1}$ (red) and 1 kVm$^{-1}$ (black). Inset: detail of area indicated by the gray box.

See also Figure S1.

electric field ~10 m above the canopy of a 35-m-tall tree can exceed 2 kVm$^{-1}$ (Figures 1B–1E and S1). Closer to the tree, around sharp leaf, needle, and branch tips, e-fields easily reach tens of kilovolts per meter (Figures 1B–1E and S1). Local e-fields can become very high under observed atmospheric conditions; the potential difference between a grounded plant and the surrounding air is often high enough to initiate ion emission by corona discharge [26, 28–31].

APGs and the e-fields surrounding all matter are relevant to biological systems; for example, bumblebees can detect e-fields arising between themselves and flowers [27], and honeybees can use their charge to communicate within the hive [32]. But beyond bees, how widespread is the ability to detect and use electrostatic forces in terrestrial organisms? Spider silk has long been known as an effective electrical insulator; indeed, it was used in the first quantitative measurements of electrostatic charge by Michael Faraday and is positioned at the bottom of the triboelectric series, where it accumulates a net negative charge [33]. Previous theoretical considerations have proposed that when silk is charged, the APG can provide sufficient coulomb force to enable ballooning and aerial suspension using electrostatic forces alone [7]. Quite surprisingly, APG is rarely invoked, let alone quantified, in conventional weather descriptors and parameters collected by weather stations. As the APG plays a role in defining e-fields surrounding vegetation, it is reasonable to surmise that if e-fields are ecologically relevant, spiders should be able to detect and respond to an e-field by changing their behavior to engage in ballooning. Here, we presented adult Linyphiid spiders (*Erigone*) with e-fields quantitatively commensurate with atmospheric conditions. Spiders were placed on a vertical strip of cardboard in the center of a polycarbonate box, limiting air movement. This box also served as an APG simulator in the form of a parallel-plate capacitor. This entire setup was situated within an acoustic isolation and Faraday cage room (3 m × 2.8 m × 2.25 m). In their natural environment, ballooning spiders take off from protruding branches,

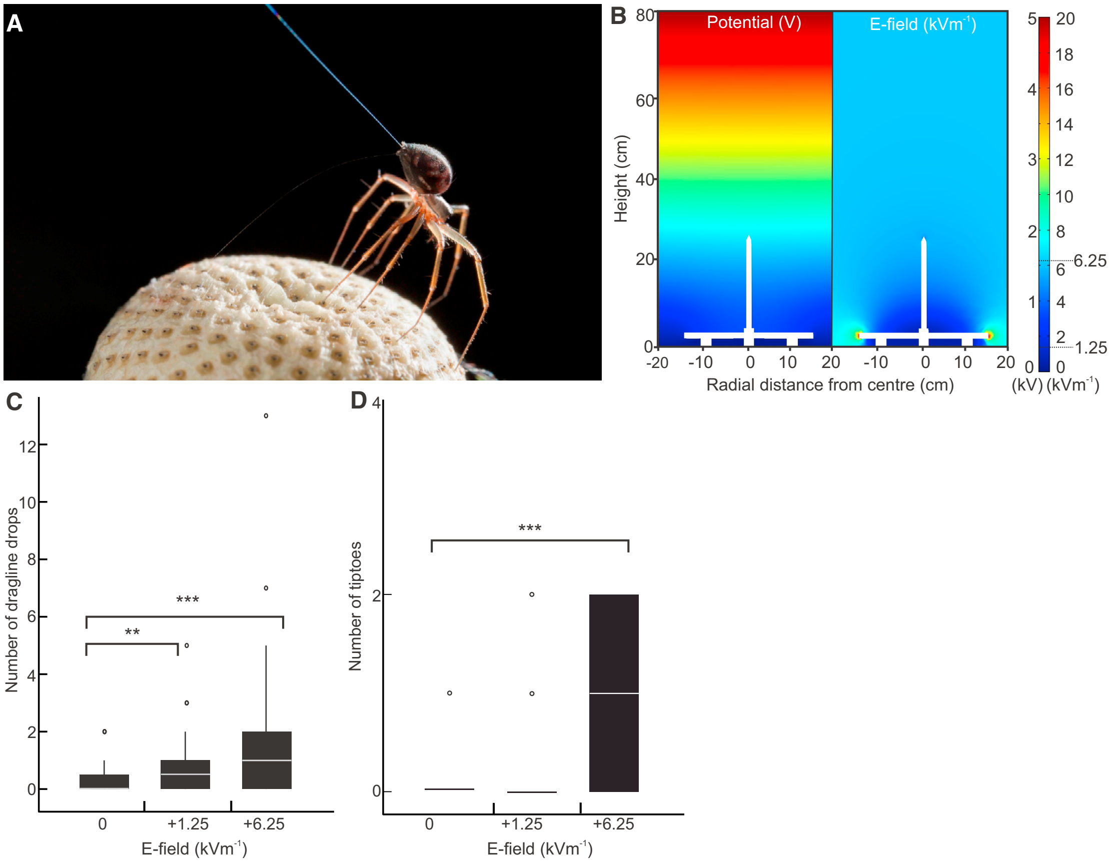


**Figure 2. Spider Ballooning Behavior**

(A) A spider showing a typical tiptoe stance.

(B) Finite element model of the electric potential (left) and e-field (right) in the behavioral arena. The electric potential is the potential energy required to move a charge from one place to another without producing any acceleration: the amount of work per unit charge. It is a scalar quantity. The electric field is a vector quantity and a force that surrounds an electric charge. It exerts either an attractive or repelling force on other charges. The base is modeled as ground with 5,000 V applied to the top plate. A water moat surrounds the takeoff site to prevent spiders escaping over ground. The water was electrically floating, not connected to ground or a voltage. The scale bar shows electric potential (left) and e-field (right). Aside from small areas around the base of the arena, the e-field is fairly uniform with a strength of 6.25 $kVm^{-1}$ (blue color indicated on the scale bar).

(C and D) Boxplots showing the (C) number of dragline drops in response to 1.25 $kVm^{-1}$, 6.25 $kVm^{-1}$, and zero-voltage control and (D) the number of tiptoes in response to 1.25 $kVm^{-1}$, 6.25 $kVm^{-1}$ and zero-voltage control (D). Significance levels: ***$p < 0.001$, **$p < 0.01$.

See also Video S1 and Table S1.

leaves, or fences. We used a non-conductive, glue-free cardboard to construct a triboelectrically neutral takeoff site. This takeoff site generates a spatially uniform and moderate e-field within the arena (Figure 2A). Vertical e-field strengths across the arena were either 0 $Vm^{-1}$ control conditions, 1.25 $kVm^{-1}$, or 6.25 $kVm^{-1}$, encompassing APG values observed in overcast, misty, and stormy weather [23], as well as e-fields around grounded trees, grasses, and flowers [6, 25, 30] (Figure 1).

There are two behavioral proxies for ballooning in spiders: the upward extension of the opisthosoma and silk extrusion, referred to as tiptoeing (Figure 2A), and dropping on a silk dragline followed by extrusion of ballooning silk [3]. Although both behaviors allow spiders to become airborne, tiptoeing exclusively precedes ballooning and is an established predictor of ballooning propensity [2]. The occurrence of these behaviors was video recorded under the different experimental treatments and subsequent analysis scored blind.

Spiders show a significant increase in ballooning in the presence of e-fields (tiptoes ΔAIC [Akaike information criterion] between full and null model 42.1, AIC 153.1 versus 195.2, d.f. = 2, $p < 10^{-6}$; dragline drops ΔAIC between full and null model 28.1, AIC 310.5 versus 282.4, d.f. = 2, $p < 10^{-6}$; Figures 2C and 2D). Significantly more dragline drops are elicited at 1.25 $kVm^{-1}$ ($Z = 2.95$; $p = 0.003$) and 6.25 $kVm^{-1}$ ($Z = 4.87$; $p < 10^{-6}$), and there is a significant increase in the number of tiptoes at 6.25 $kVm^{-1}$ ($Z = 4.03$; $p < 10^{-6}$) (Table S1). The observed change in spider behavior establishes that they can detect APG-like e-fields. Moreover, the spider's unlearned response to e-fields is to engage in ballooning, and, on becoming airborne, switching the e-field on and off results in the spider moving upward (on) or downward (off) (Video S1).

The behavioral experiments demonstrate that spiders can detect e-fields, but what is the sensory basis of spider e-field

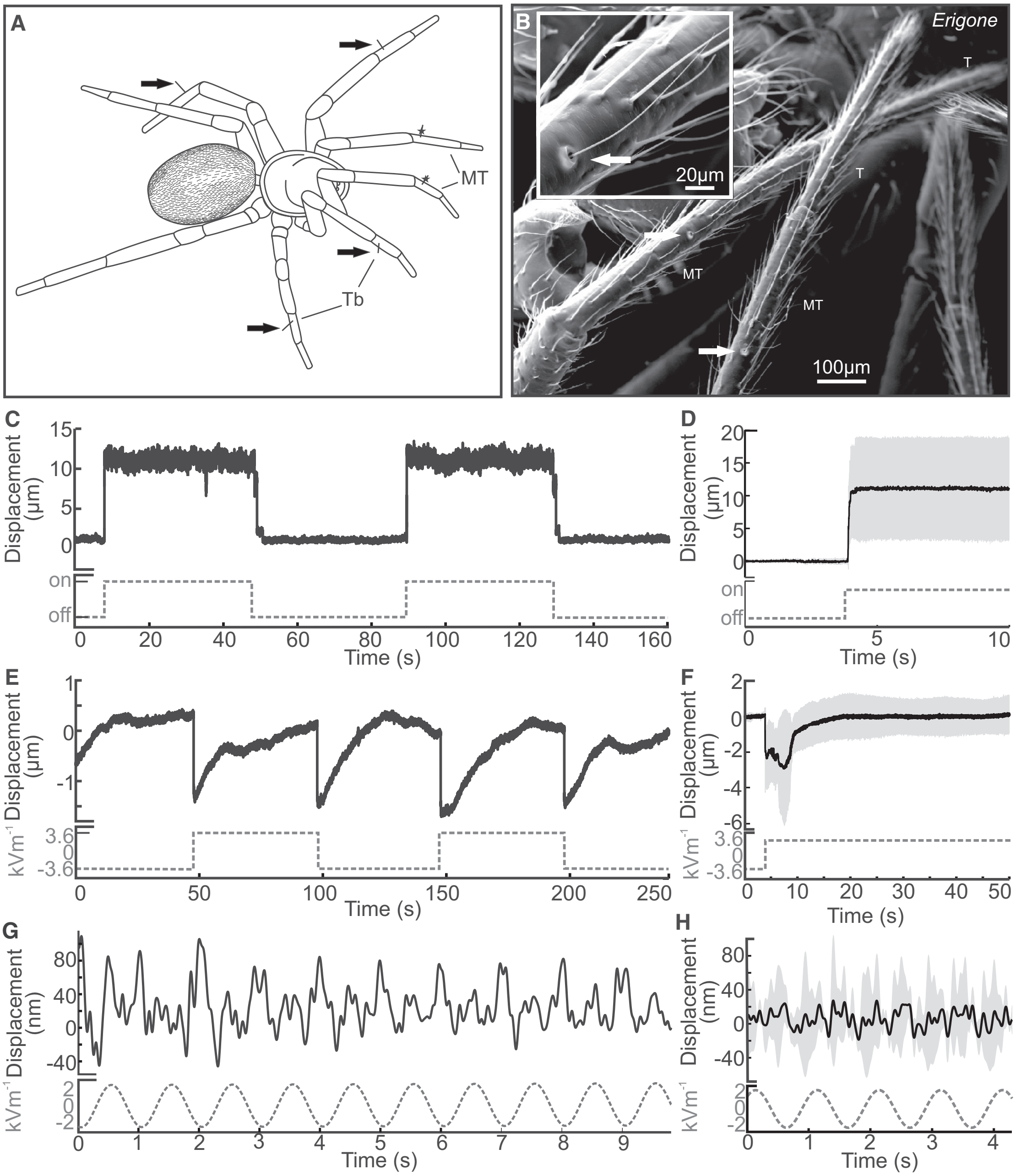


**Figure 3. Mechanical Displacement of Spider Trichobothria**

Trichobothria in *Erigone*.

(A) Diagram of a spider illustrating locations of metatarsal trichobothria and locations for non-contact laser Doppler vibrometry measurement (stars).

(B) Scanning electron microscopy image of adult male *Erigone* metatarsi and trichobothria, with a close-up view of trichobothrium (inset). Arrows point to the base of trichobothrium. MT, metatarsus; T, tarsus.

(C–H) Displacement of trichobothria in response to 0.5 $ms^{-1}$ air flow (C and D), pseudo-DC efield (E and F), and 1 Hz sine e-field (G and H) measured using laser Doppler vibrometry (LDV). (C), (E), and (G) show single traces, and (D), (F), and (H) show the mean (black) and SD (gray). n = 6 (D), n = 5 (F), and n = 4 (H). Gray dashed lines indicate the stimulus.

detection? In bumblebees, mechanosensory hairs are the putative electroreceptors sensitive to e-fields [34]. Arachnids have mechanosensory hairs known as trichobothria (Figures 3A and 3B). Much is known about their mechanical and neural response to medium flows (air and water) [35, 36]; they are exquisitely sensitive, detecting air motion close to thermal noise [37], they detect sound [38], and they are omnidirectional [39]. Early studies using electrostatic actuation as a tool to investigate trichobothria mechanics indicate that they may also be sensitive to e-fields [39–41].

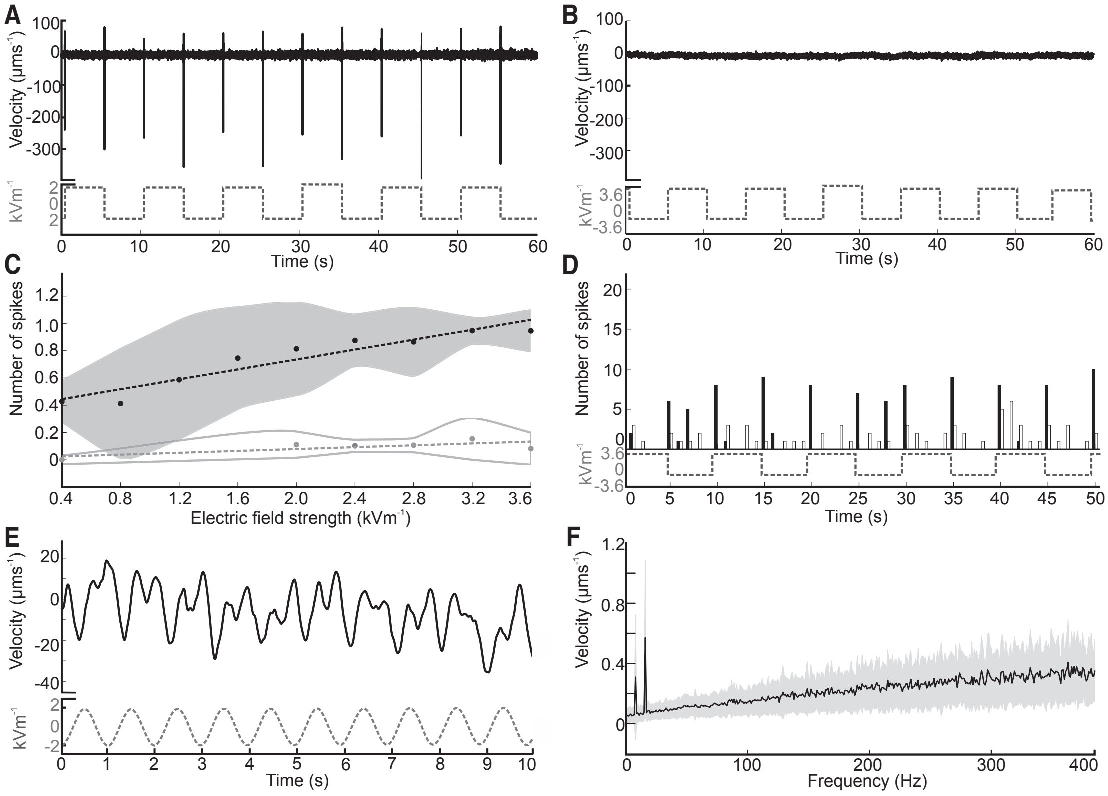


**Figure 4. Velocity of Trichobothria Motion in Response to E-Fields**

(A) Transient changes in velocity of a trichobothrium (black, solid line) in response to a 2 kVm$^{-11}$ e-field oscillating at 0.1 Hz (gray, dashed line).

(B) Transient changes in velocity of a metatarsal spine (black, solid line) in response to a 3.6 kVm$^{-11}$ e-field oscillating at 0.1 Hz (gray, dashed line).

(C) Spike rate (as seen in A and B) of trichobothria (black; n = 8; ±SD) and metatarsal spine (gray; n = 4; ±SD) across a range of e-field strengths. Spike rate was measured as the ratio between the total number of zero crossing of the e-field stimulus to the number of spikes coincident (within 25 ms) of stimulus zero crossings.

(D) Histogram (binned every 25 ms) of the number of velocity spikes of the trichobothria (black; n = 8) and metatarsal spines (white; n = 4) in response to a 0.1 Hz square wave. The dashed gray line shows stimulus recording.

(E) Velocity of a trichobothrium (black, solid line) in response to an e-field oscillating at 1 Hz (gray, dashed line).

(F) Frequency response (FFT) of trichobothria (black; n = 6; ±SD) in response to a 1 Hz sine wave e-field.

We tested the mechanical response of trichobothria on the front metatarsus to both air flow (0.5 ms$^{-1}$) and e-fields using laser Doppler vibrometry (LDV). Pseudo-direct current (DC) electrical stimuli with 0.1 Hz and 0.01 Hz square waves were used to simulate a static deflection and rapid change in e-field, as happens when charged clouds pass overhead (Figure 1). Also, a 1 Hz sine wave was used to investigate the response to slowly changing e-fields. The response to air flow, a stimulus long established to deflect trichobothria, was also measured for comparison. Trichobothria are displaced in different ways by DC air flow and DC e-fields (Figures 3C–3F). In response to air flow, trichobothria are statically displaced for the duration of stimulus presentation, a tonic response. In contrast, displacement to e-fields is maximal at the transient switch in voltage, decreasing back to the baseline over a period of around 30 s, a phasi-tonic response. Here, the direction of trichobothria displacement is independent of stimulus polarity; both positive-to-negative and negative-to-positive stimulus transitions produce displacement in the same direction, a response indicative of induction charging where forces are always attractive regardless of stimulus polarity. Notably, the different types of mechanical response generated by air movement and e-fields suggest that wind and electric field detection can be differentiated despite sharing a common peripheral receptor. The trichobothrium is also displaced in response to a 1 Hz sine wave (Figures 3G and 3H), showing that they mechanically respond to slowly varying e-fields, as well as to rapid changes in potential. Here, the frequency response of the trichobothrium is twice that of the stimulus (Figures 4E and 4F); each zero crossing of the stimulus generates a change in the direction of displacement of the trichobothrium, providing additional evidence of electrostatic induction. The response of trichobothria, measured as the number of times the velocity spikes (Figure 4A), scales linearly with e-field strength within the range measured (3.6–0.4 kVm$^{-1}$). No response above instrumentation noise (typically 2–10 pm) was elicited from spines (Figures 4B and 4C). The measurement of tibial spines is a useful control allowing the exclusion of non-stimulus specific air motion, electrical crosstalk, or the motion of the entire animal as potential drivers of the responses measured from trichobothria. Hence, the trichobothria's mechanical response can be

considered to result from forces applied to them by the electric field. Such sensitivity to ambient e-field strength is compatible with the notion that spider trichobothria can work as electromechanical receptors. The neuroethology of trichobothria in response to e-fields needs further characterization, to add to the detailed knowledge of their response to medium flows.

This is the first demonstration of aerial electroreception in spiders and in arthropods beyond Apidae. The phylogenetic distance between spiders and bees indicates that aerial electroreception could be widespread among the Arthropoda. Consequently, the electromechanical sensitivity of hair structures present in bumblebees and spiders indicates a possible dual function, as medium flow sensors and electroreceptors. The hypothesis thus emerges that the mechanosensory hairs of many arthropod species may exhibit the additional function of aerial electroreception.

The present evidence shows that the APG and resulting electrostatic forces are sufficient to elicit ballooning, yet they may not always be necessary. Aerodynamic drag associated with light wind and electrostatic forces can work in synergy to facilitate ballooning. As a result of this work, we propose that the APG serves at least three functions: an indicator of meteorological conditions, an informational trigger, and a physical driving force enabling ballooning. Several mechanistic questions now emerge, pertaining to the dielectric characteristics of ballooning silk and whether altitude control and navigation take place. Future work needs to disentangle the complex interplay between animal behavior and variations in the APG. Inclusion of the APG as a meteorological parameter has the potential to provide better predictions of dispersal events and the distribution of spider populations.

Understanding the mechanisms that underpin dispersal is crucial for describing biomass and gene flow, population dynamics, species distributions, and ecological resilience to stochastic changes. It is therefore of great importance for global ecology. Spiders are a powerful source of biological control, consuming 400–800 million tons of biomass globally each year [42], significantly impacting the composition and diversity of ecosystems [43]. The terrestrial biological world has evolved within the APG and the use of e-fields in dispersal could extend beyond ballooning spiders to those species of caterpillar (Lepidoptera) and spider mite (Trombidiformes) that also disperse aerially [2], as well as plant propagules. As ballooning arthropods constitute a proportion of significant seasonal bioflows [8], studying the role of atmospheric electricity and its detection by arthropods has implications for predicting the transport of nutrients, pathogens, agricultural pests [44–46], and their predators between ecosystems and biomes [8].

## STAR★METHODS

Detailed methods are provided in the online version of this paper and include the following:

- KEY RESOURCES TABLE
- CONTACT FOR REAGENT AND RESOURCE SHARING
- EXPERIMENTAL MODEL AND SUBJECT DETAILS
- METHOD DETAILS
  - Behavioral experiment set up
  - Protocol for behavioral experiments
  - Trichobothria mechanics
  - Electric field models
- QUANTIFICATION AND STATISTICAL ANALYSIS
- DATA AND SOFTWARE AVAILABILITY

## SUPPLEMENTAL INFORMATION

Supplemental Information includes one figure, one table, and one video and can be found with this article online at https://doi.org/10.1016/j.cub.2018.05.057.

A video abstract is available at https://doi.org/10.1016/j.cub.2018.05.057#mmc4.

## ACKNOWLEDGMENTS

E.L.M. would like to thank Andrew Mason for encouraging the collection of pilot data. Both E.L.M. and D.R. thank four anonymous reviewers for constructive comments that allowed improvement to our manuscript. E.L.M. and D.R. were supported by grants from the UK BBSRC (BB/M011143/1) and European Research Commission (ERC ADG 743093).

## AUTHOR CONTRIBUTIONS

Conceptualization, E.L.M.; Methodology, E.L.M. and D.R.; Investigation, E.L.M; Writing – Original Draft, E.L.M.; Writing – Review & Editing, E.L.M. and D.R.; Resources, D.R.

## DECLARATION OF INTERESTS

The authors declare no competing interests.

Received: September 19, 2017
Revised: May 4, 2018
Accepted: May 18, 2018
Published: July 5, 2018

## REFERENCES

1. Glick, P.A. (1939). The distribution of insects, spiders, and mites in the air. United States Department of Agriculture, technical bulletin no. 673. https://naldc.nal.usda.gov/download/CAT86200667/PDF.
2. Bell, J.R., Bohan, D.A., Shaw, E.M., and Weyman, G.S. (2005). Ballooning dispersal using silk: world fauna, phylogenies, genetics and models. Bull. Entomol. Res. *95*, 69–114.
3. Weyman, G.S. (1993). A review of the possible causative factors and significance of ballooning in spiders. Ethol. Ecol. Evol. *5*, 279–291.
4. Humphrey, J.A.C. (1987). Fluid mechanic constraints on spider ballooning. Oecologia *73*, 469–477.
5. Reynolds, A.M., Bohan, D.A., and Bell, J.R. (2007). Ballooning dispersal in arthropod taxa: conditions at take-off. Biol. Lett. *3*, 237–240.
6. Wilson, C.T.R. (1903). Atmospheric electricity. Nature *68*, 102–104.
7. Gorham, P.W. (2013). Ballooning spiders: the case for electrostatic flight. arXiv, arXiv:1309.4731v, http://arxiv.org/abs/1309.4731v.
8. Hu, G., Lim, K.S., Horvitz, N., Clark, S.J., Reynolds, D.R., Sapir, N., and Chapman, J.W. (2016). Mass seasonal bioflows of high-flying insect migrants. Science *354*, 1584–1587.
9. Blackwall, J. (1830). Mr Murray's paper on the aerial spider. Mag. Nat. Hist. J. Zool. Bot. Mineral. Geol. Meteorol. *2*, 116–413.
10. Keynes, R.D., ed. (2000). Charles Darwin's Zoology Notes & Specimen Lists from H.M.S. Beagle (Cambridge University Press), pp. 106–108.
11. Suter, R.B. (1999). An aerial lottery: the physics of ballooning in a chaotic atmosphere. J. Arachnol. *27*, 281–293.
12. Schneider, J.M., Roos, J., Lubin, Y., and Henschel, J.R. (2001). Dispersal of *Stegodyphus dumicola* (Araneae eresidae): they do balloon after all! J. Arachnol. *29*, 114–116.

13. Sparkes, J., and Holland, C. (2017). Analysis of the pressure requirements for silk spinning reveals a pultrusion dominated process. Nat. Commun. *8*, 594.
14. Weyman, G.S., Sunderland, K.D., and Jepson, P.C. (2002). A review of the evolution and mechanisms of ballooning by spiders inhabiting arable farmland. Ethol. Ecol. Evol. *14*, 307–326.
15. Greenstone, M.H. (1990). Meteorological determinants of spider ballooning: the roles of thermals vs. the vertical windspeed gradient in becoming airborne. Oecologia *84*, 164–168.
16. Tolbert, W.W. (1977). Aerial dispersal behavior of two orb weaving spiders. Psyche. Aust. J. Entomol. *84*, 13–27.
17. Vugts, H.F., and Van Wingerden, W.K.R.E. (1976). Meteorological aspects of aeronautic behaviour of spiders. Oikos *27*, 433–444.
18. Yeargan, K.V. (1975). Factors influencing the aerial dispersal of spiders (Arachnida: Araneida). J. Kans. Entomol. Soc. *48*, 403–408.
19. Richter, C.J.J. (1970). Aerial dispersal in relation to habitat in eight wolf spider species (Pardosa, Araneae, Lycosidae). Oecologia *5*, 200–214.
20. Bishop, L. (1990). Meteorological aspects of spider ballooning. Environ. Entomol. *19*, 1381–1387.
21. Simonneau, M., Courtial, C., and Pétillon, J. (2016). Phenological and meteorological determinants of spider ballooning in an agricultural landscape. C. R. Biol. *339*, 408–416.
22. Rycroft, M.J., Nicoll, K.A., Aplin, K.L., and Harrison, R.G. (2012). Recent advances in global electric circuit coupling between the space environment and the troposphere. J. Atmos. Solar-Terrestrial Physiol. *90–91*, 198–211.
23. Bennett, A. (2007). Measurement of atmospheric electricity during different meteorological conditions. PhD thesis (University of Reading).
24. Feynman, R.P., Leighton, R.B., and Sands, M.L. (1964). The Feynman Lectures on Physics: Mainly Electromagnetism and Matter (Addison-Wesley).
25. Clarke, D., Morley, E., and Robert, D. (2017). The bee, the flower, and the electric field: electric ecology and aerial electroreception. J. Comp. Physiol. A Neuroethol. Sens. Neural Behav. Physiol. *203*, 737–748.
26. Borra, J.-P. (1995). Étude de la formation des décharges électriques de pointe dans la nature: analyse des produits gazeux et particulaire en conditions controlées. PhD thesis (Paris VI University).
27. Clarke, D., Whitney, H., Sutton, G., and Robert, D. (2013). Detection and learning of floral electric fields by bumblebees. Science *340*, 66–69.
28. Aubrecht, L., Stanek, Z., and Koller, J. (2001). Corona discharge on coniferous trees-spruce and pine. Europhys. Lett. *53*, 304–309.
29. Aubrecht, L., Koller, J., and Stanek, Z. (2000). Onset voltages of atmospheric corona discharges on plants. Czeckoslovak J. Physiol. *50*, 313–318.
30. Borra, J.-P., Roos, R., Renard, D., Lazar, H., Goldman, A., and Goldman, M. (1997). Electrical and chemical consequences of point discharges in a forest during a mist and a thunderstorm. J. Phys. D Appl. Phys. *30*, 84–93.
31. Bent, R.B., Collin, H.L., Hutchinson, W.C.A., and Chalmers, J.A. (1965). Space charges produced by point discharge from trees during a thunderstorm. J. Atmos. Terr. Phys. *27*, 67–72.
32. Greggers, U., Koch, G., Schmidt, V., Dürr, A., Floriou-Servou, A., Piepenbrock, D., Göpfert, M.C., and Menzel, R. (2013). Reception and learning of electric fields in bees. Proc. Biol. Sci. *280*, 20130528.
33. Vollrath, F., and Edmonds, D. (2013). Consequences of electrical conductivity in an orb spider's capture web. Naturwissenschaften *100*, 1163–1169.
34. Sutton, G.P., Clarke, D., Morley, E.L., and Robert, D. (2016). Mechanosensory hairs in bumblebees (*Bombus terrestris*) detect weak electric fields. Proc. Natl. Acad. Sci. USA *113*, 7261–7265.
35. Barth, F.G. (2002). Trichobothria - the measurement of air movement. A Spider's World (Springer), pp. 85–111.
36. Reißland, A., and Görner, P. (1985). Trichobothria. In Neurobiology of Arachnids, F. Barth, ed. (Springer), pp. 138–161.
37. Humphrey, J.A.C., Barth, F.G., Reed, M., and Spak, A. (2003). The physics of arthropod medium-flow sensitive hairs: biological models for artificial sensors. In Sensors and Sensing in Biology and Engineering, F.G. Barth, J.A.C. Humphrey, and T.W. Secomb, eds. (Springer), pp. 129–144.
38. Shamble, P.S., Menda, G., Golden, J.R., Nitzany, E.I., Walden, K., Beatus, T., Elias, D.O., Cohen, I., Miles, R.N., and Hoy, R.R. (2016). Airborne acoustic perception by a jumping spider. Curr. Biol. *26*, 2913–2920.
39. Reißland, A., and Görner, P. (1978). Mechanics of trichobothria in orb-weaving spiders (Agelenidae, Araneae). J. Comp. Physiol. A Neuroethol. Sens. Neural Behav. Physiol. *123*, 59–69.
40. Hoffmann, C. (1967). Bau und Funktion der Trichobothrien von *Euscorpius carpathicus L*. Zeitschrift Für Vergleichende Physiol. *54*, 290–352.
41. Görner, P. (1965). Mehrfach innervierte Mechanorezeptoren (Trichobothrien) bei Spinnen. Naturwissenschaften *437*.
42. Nyffeler, M., and Birkhofer, K. (2017). An estimated 400-800 million tons of prey are annually killed by the global spider community. Naturwissenschaften *104*, 30.
43. Schmitz, O.J., and Suttle, K.B. (2001). Effects of top predator species on direct and indirect interactions in a food web. Ecology *82*, 2072–2081.
44. Scarpino, V., Reyneri, A., Vanara, F., Scopel, C., Causin, R., and Blandino, M. (2015). Relationship between European Corn Borer injury, *Fusarium proliferatum* and *F. subglutinans* infection and moniliformin contamination in maize. F. Crop. Res. *183*, 69–78.
45. Jeppson, L.R., Keifer, H.H., and Baker, E.W. (1975). Mites Injurious to Economic Plants (University of California Press).
46. Muralidharan, K., and Pasalu, I.C. (2006). Assessments of crop losses in rice ecosystems due to stem borer damage (Lepidoptera: Pyralidae). Crop Prot. *25*, 409–417.
47. R Core Team (2016). R: a language and environment for statistical computing. (R Foundation for Statistical Computing). https://www.R-project.org/.
48. Bates, D., Maechler, M., Bolker, B., and Walker, S. (2015). Fitting linear mixed-effects models using lme4. J. Stat. Softw. *67*, 1–48.
49. Woolley, C.F., Thomas, G., Hutchings, L., Goodacre, S., Hewitt, G.M., and Brooks, S.P. (2007). A novel trap to capture ballooning spiders. J. Arachnol. *35*, 307–312.

# STAR★METHODS

## KEY RESOURCES TABLE

| REAGENT or RESOURCE | SOURCE | IDENTIFIER |
|---|---|---|
| Deposited Data | | |
| Experimental data and COMSOL models | This paper | https://doi.org/10.17632/8vpyymcrt4.1 |
| Experimental Models: Organisms/Strains | | |
| *Erigone* spp. | Langford School of Veterinary Sciences, Bristol, UK | N/A |
| Software and Algorithms | | |
| COMSOL Multiphysics 5.3a | COMSOL Multiphysics | https://www.comsol.com/ |
| MATLAB 2014a | Mathworks | https://www.mathworks.com |
| R Studio version 0.99.893 | [47] | https://www.rstudio.com/ |
| Ime4 package for R | [48] | https://cran.r-project.org/web/packages/lme4/index.html |
| PSV version 9.0 | Polytec | https://www.polytec.com/eu/ |

## CONTACT FOR REAGENT AND RESOURCE SHARING

Further information and requests for resources and reagents should be directed to and will be fulfilled by the Lead Contact, Erica Morley (erica.morley@bristol.ac.uk).

## EXPERIMENTAL MODEL AND SUBJECT DETAILS

*Erigone* spp. were caught by balloon trap [49] at the University of Bristol School of Veterinary Sciences, Langford between July and October 2016. They were housed individually at 17°C on 12:12 hr light dark cycles until the start of experiments. Adult males and females were identified by examining palps and epigyne under a light microscope.

## METHOD DETAILS

### Behavioral experiment set up

Behavioral responses of adult *Erigone* to different electric field strengths were observed under experimental treatments with a repeated-measures, randomized block design. The experimental arena comprised a polycarbonate box (0.9 m × 0.9 m × 0.9 m) to limit air motion, with a door on one side for access. An aluminum plate (0.8 m × 0.8 m) was attached inside the top of the box and an identical aluminum plate was positioned on the bottom of the box to give a plate separation of 0.8 m. The plates were connected to a high voltage power supply (PS350; Stanford Research Systems, Sunnyvale, CA, USA), with one plate electrically grounded and the other connected to either 0 V, 1000 V or 5000 V to give electric field strengths of 0 $Vm^{-1}$, 1.25 $kVm^{-1}$ or 6.25 $kVm^{-1}$ respectively. A plastic dish (37 cm diameter) with a glue-free cardboard strip oriented vertically (25 cm, 1 m at the base tapering to 2 mm at the tip) was positioned in the center of the box. The takeoff site was surrounded by shallow water to limit the escape of spiders. The water was not connected to ground or voltage and was electrically floating. The entire setup was situated on an anti-vibration table (Newport RS4000; Irvine, CA, USA) within an acoustic isolation and Faraday cage room (2.8 m × 3 m × 2.25 m). Temperature and humidity levels were monitored throughout experiments (21.2° ± 0.9; 50.5% RH ± 5.4).

Spider behavior was subsequently assessed by video analysis. To minimize experimenter bias, videos were scored blind. The number of tiptoe events and dragline drops were recorded during the 2-min treatment. Tiptoes were defined as holding the typical tiptoe stance (Figure 2A) for at least 3 s. Attempts that did not meet this criterion were not counted.

### Protocol for behavioral experiments

In each trial, spiders were placed individually at the top of the takeoff site. They were given an initial 5 min settling period, following which the treatment was turned on and their behavior filmed (Canon EOS 700D, Canon Macro EF 100 mm 1:2.8 L IS USM; Canon, Tokyo, Japan) for 2 min. After 2 min, the treatment was switched off and the filming stopped. The spider was then removed and put back in its vial. Each spider was subjected to 3 treatments in a randomized order. Only 1 treatment was tested per animal each day and trials were completed across consecutive days. Between each trial the takeoff site was wiped with a cloth containing 70% ethanol and allowed to dry to remove silk and possible chemical cues left by the spider in the previous trial. Any residual charge was neutralised between each trial using an anti-static gun (Zerostat 3; Milty), and monitored using a custom built electroscope. The effect of electric field strength was tested using positive voltages applied to the top plate (1000 V, 5000 V) and a control (0 V).

The voltage was applied to the top plate with an electrically grounded bottom plate. To control for experimenter disturbance, the 0 V control treatment was carried out in the same way as the voltage treatments; the power supply was physically disconnected from the behavioral set up before the trial began, and the power supply was manually switched on and off in the same way as the voltage treatments to cause the same level of disturbance as in other treatments, but without any applied voltage.

## Trichobothria mechanics

The mechanical response of trichobothria to electrostatic fields was measured in adult male *Erigone* using laser Doppler vibrometry (LDV) (Polytec PSV 400, Polytec, Waldbronn, Germany). Spiders were anaesthetised using $CO_2$ and affixed by the dorsal prosoma to a wooden stick (tip diameter 1mm) using liquid latex. The opisthosoma, legs and palps were all immobilised using liquid latex and the spider was positioned so that the metatarsal trichobothrium on one of the first pair of legs was within focus of the laser beam and coaxial camera, using a close-up attachment (PSV-A-410; Polytec, Waldbronn, Germany). Parallel aluminum plates (15 cm × 11 cm) were positioned above and below the spider, 10cm apart. The top plate was connected to a voltage source (Agilent 33120A; custom built high voltage amplifier) and the bottom plate was electrically grounded. The entire set up was placed on an anti-vibration table (TMC 784-443-12R; Technical Manufacturing, Peabody, MA, USA) within an anechoic chamber (2.25 m × 2.7 m × 2.6 m) electrically isolated by Faraday caging.

The pseudo-DC voltage comprised a square wave of 0.01 Hz at 360 V peak-to-peak (pp) (3.6 $kVm^{-1}$), to allow for a clear displacement measurement. The velocity of trichobothria response to a 0.1 Hz square wave in voltages steps from 360 V (3.6 $kVm^{-1}$) to 40 V (0.4 $kVm^{-1}$) was also measured, along with the displacement and velocity response to a 1 Hz sine wave at 200 V (2 $kVm^{-1}$). Measurements were made in the time domain, digitized via an on-board data acquisition card (National Instruments PCI- 6110) and subsequently analyzed by using PSV software (Polytec version 9.0). Data analysis was carried out in MATLAB 2014a (Mathworks, Natick, MA, USA).

## Electric field models

Finite element models of the APG around trees and e-fields within the experimental arena were generated using COMSOL Multiphysics (COMSOL 5.3a, Stockholm, Sweden). The models of the APG-tree interactions were produced by modeling the APG during unsettled weather with an electric field strength of either 1 $kVm^{-1}$ or 4 $kVm^{-1}$. Here, electrical ground was beneath 5 m of soil which had an electrical boundary with the tree. The electric potential was modeled as a plate above the air. To model the experimental arena a cardboard takeoff site (with appropriate material properties) was generated inside a volume of air of the same dimension as the arena. Below the takeoff site a dish of water was modeled. The bottom surface was held at ground potential while the top plate of the arena was set at 5000 V, the maximum used in behavioral experiments. The cardboard blade and water dish were left electrically floating, as they were in the arena itself.

# QUANTIFICATION AND STATISTICAL ANALYSIS

The ballooning behavior data was analyzed using generalized linear mixed models (GLMM) using the lme4 [48] package in R Studio version 0.99.893 [47]. All data was in the form of counts and a total of 36 animals (n = 36, 20 males and 16 females) were used in the behavioral study. Each animal was subjected to all e-field treatments (0 V/m, 1.25 V/m and 6.25 V/m), therefore the analysis needed to include spider identification as a factor to incorporate the repeated-measures design into the statistical analysis. Data was bounded at 0 (count data) and due to singularity, models with different intercepts and slopes did not converge, a simple random effects model was therefore used with a common slope but different intercepts. To test for the effect of voltage treatment on behavior a full model was compared to a null model with treatment removed using ANOVA and ΔAIC scores. The random factor in each test was the individual spider (1|spider identification) while the fixed factor was the voltage treatments tested. Tests used Poisson error and a log link function. Median and interquartile ranges for the data are shown in Figure 2 along with significance values and GLMM outputs are described in Table S1.

# DATA AND SOFTWARE AVAILABILITY

Experimental data and COMSOL models are made available through Mendeley Data and can be accessed at https://doi.org/10.17632/8vpyymcrt4.1.

Current Biology, Volume *28*

## Supplemental Information

# Electric Fields Elicit Ballooning in Spiders

**Erica L. Morley and Daniel Robert**

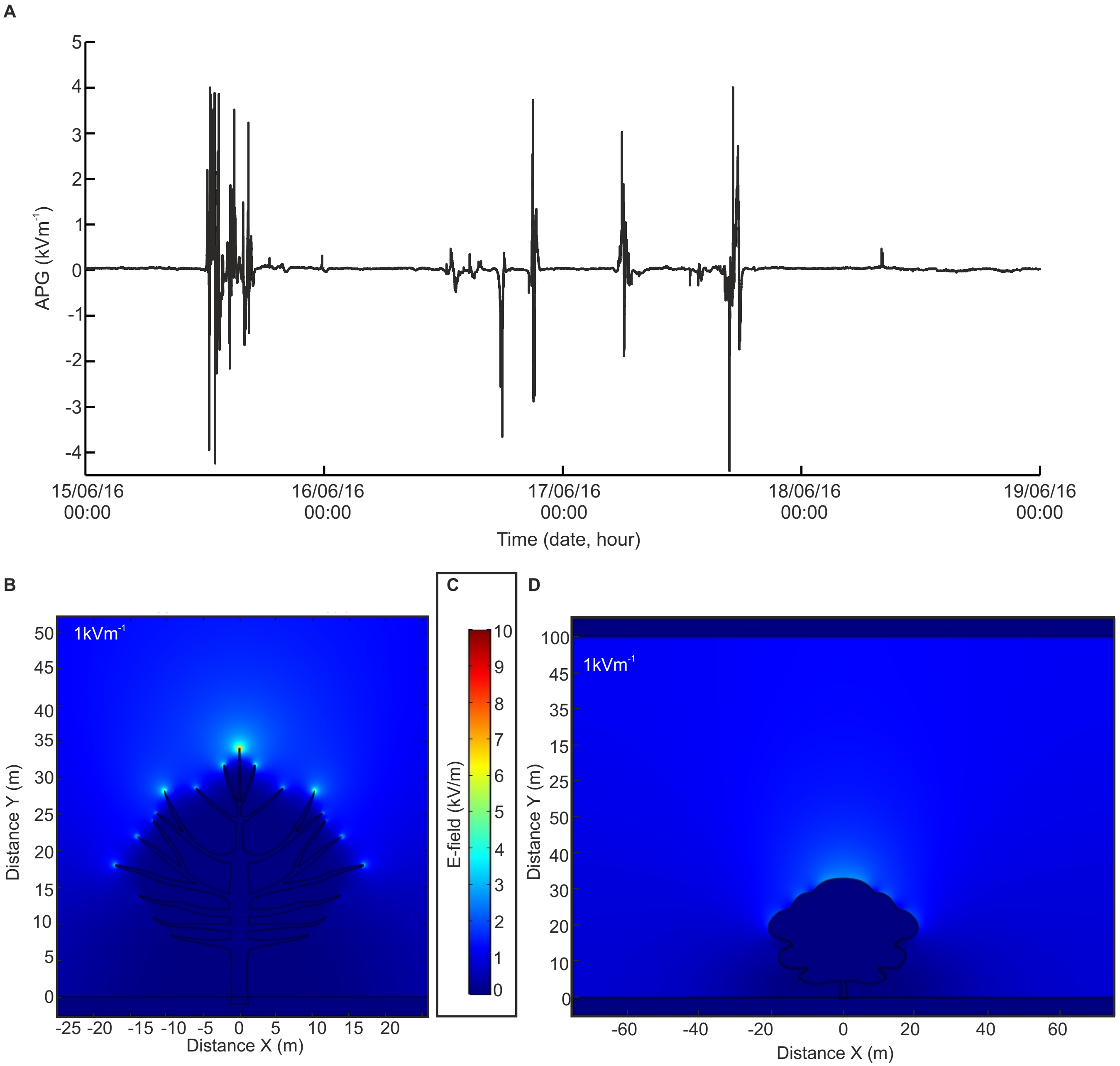


**Figure S1. Further electric field quantifications, related to Figure 1**

A) Atmospheric potential gradient (APG) measured across 4 days using a field mill (Chillworth JCI131) at the University of Bristol School of Veterinary Sciences, Langford. B) FEA model detailing the e-field around geometrically sharp tree branches in an APG strength of $1kVm^{-1}$.C) Scale bar for (B) and (D). D) FEA model of e-field enhancement around a geometrically domed oak tree in an APG strength of $1kVm^{-1}$. The scale in figure S1 B-D are the same as the scale in figure 1B-D, for comparison.

| Distribution | Fixed factor | Level | Coefficient estimate | Standard error | Z value | p value |
|---|---|---|---|---|---|---|
| Tiptoes | | | | | | |
| Poisson | Field strength (reference $0Vm^{-1}$) | $1.25Vm^{-1}$ | 1.25 | 0.8 | 1.565 | 0.118 |
| | | $6.25Vm^{-1}$ | 2.91 | 0.72 | 4.025 | <0.0001*** |
| Dragline drops | | | | | | |
| Poisson | Field strength (reference $0Vm^{-1}$) | $1.25Vm^{-1}$ | 1.04 | 0.35 | 2.952 | 0.003** |
| | | $6.25Vm^{-1}$ | 1.61 | 0.33 | 4.873 | <0.0001*** |

**Table S1. GLMM statistical analysis, related to Figure 2**

Results of GLMM fits to ballooning behaviour data. All models had spider identification number as a random factor with varied intercept and the same slope (1|spider identification) to prevent pseudoreplication of repeated measurements on the same animal.
